# Resultados da transferência do latíssimo do dorso associada ao uso de enxerto de tendão autólogo da fáscia lata nas lesões posterossuperiores irreparáveis do manguito rotador

**DOI:** 10.1055/s-0046-1819620

**Published:** 2026-04-22

**Authors:** Ricardo Makoto Okamoto, Guilherme do Val Sella, Luciana Andrade da Silva, Thomas Yogo Mendonça Alves, Alberto Naoki Miyazaki

**Affiliations:** 1 Grupo de Cirurgia de Ombro, Departamento de Ortopedia e Traumatologia, Faculdade de Ciências Médicas da Santa Casa de São Paulo, São Paulo, SP, Brasil

**Keywords:** ferimentos e lesões, manguito rotador, transferência tendinosa, transplante autólogo, rotator cuff, tendon transfer, transplantation, autologous, wounds and injuries

## Abstract

**Objetivo:**

Avaliar os resultados funcionais dos pacientes submetidos à transferência do latíssimo do dorso reforçado com um enxerto autólogo de fáscia lata retirado da coxa contralateral à lesão no tratamento das lesões posterossuperiores irreparáveis do manguito rotador (LPSIMRs). O objetivo secundário foi avaliar as complicações da retirada do enxerto.

**Métodos:**

Avaliação funcional retrospectiva de 17 pacientes (8 homens e 9 mulheres) com média de idade de 62,6 (54–73) anos e seguimento pós-operatório médio de 21,2 (12–45) meses, sendo comparados o University of California, Los Angeles (UCLA) e a amplitude de movimento (ADM) dos períodos pré e pós-operatório. Foram avaliados também se houve perda de força de abdução do quadril doador do enxerto e possíveis complicações associadas. As correlações entre as variáveis foram determinadas pelo coeficiente de Pearson, com valores de *p* ≤ 0,05.

**Resultados:**

A pontuação média UCLA do período pós-operatório foi de 27,9 (14–33) pontos com melhora de 16,7 pontos da média pré-operatória. Houve melhora das ADMs com os seguintes valores finais: elevação 27,9°; rotação lateral (RL) 133°; rotação medial (RM) em T9, todos com *p* < 0,05. Em relação ao local da retirada do enxerto, nenhum paciente apresentou sinais de infecção, dois se queixaram de dor na ferida operatória e um apresentou pequena hérnia incisional.

**Conclusão:**

A técnica realizada foi eficaz no tratamento de LPSIMRs, com 70,6% de resultados satisfatórios de acordo com a pontuação UCLA e melhora da ADM. O uso do enxerto autólogo é uma opção viável com baixos índices de complicações, tanto no ombro quanto no quadril.

## Abstract (en)

**Objective:**

To evaluate the functional outcomes of latissimus dorsi transfer reinforced with an autologous fascia lata graft harvested from the thigh contralateral to the injury to treat irreparable posterosuperior rotator cuff tears (IPSRCTs). The secondary objective was to assess complications related to graft harvesting.

**Methods:**

The present retrospective functional evaluation involved 17 patients (8 men and 9 women), with a mean age of 62.6 (54–73) years and a mean postoperative follow-up period of 21.2 (12–45) months. We compared pre- and postoperative University of California, Los Angeles (UCLA) scores and range of motion (ROM), assessed donor-site hip abduction strength and recorded potential associated complications. The determination of correlations between variables used Pearson's coefficient, with *p* ≤ 0.05 considered statistically significant.

**Results:**

The mean postoperative UCLA score was 27.9 (14–33) points, representing an improvement of 16.7 points compared to the preoperative mean. Range of motion improved significantly ( *p* < 0.05), with the following final values: elevation, 27.9°; lateral rotation (LR), 133°; medial rotation (MR) at T9, all with *p* < 0.05. No patient presented signs of infection at the graft harvest site; two subjects reported pain in the surgical wound, and one developed a small incisional hernia.

**Conclusion:**

This technique was effective in treating IPSRCTs, achieving a 70.6% satisfactory outcome based on the UCLA score and showing significant improvement in ROM. The use of an autologous graft represents a viable option with low complication rates, both at the shoulder and hip.

## Introdução

As lesões do manguito rotador podem ser causa de comprometimento funcional grave e dores incapacitantes nos ombros dos pacientes acometidos, sendo necessário o tratamento cirúrgico. 1 Dentre essas, as lesões irreparáveis do manguito rotador (LIMRs) são um desafio para os especialistas em cirurgia de ombro. Diversas técnicas cirúrgicas foram descritas para melhorar a dor e a função do ombro, tais como as artroplastias reversas, 2 a reconstrução da cápsula superior 3 4 e as transferências tendíneas, que podem incluir a do tendão do músculo trapézio 5 ou a do tendão latíssimo do dorso (TLD), 6 7 8 9 10 11 12 13 podendo estes tendões serem reforçados com enxertos tendíneos homólogos e autólogos. Pogorzelski et al. 14 utilizaram enxerto homólogo de tendão de Aquiles como reforço tendíneo nas transferências do TLD para o tratamento de 16 pacientes com LIMRs. Bouchard et al. 15 avaliaram, através de uma revisão sistemática, os resultados e complicações das transferências tendíneas no reparo das LIMRs, sendo mais comum o uso do TLD.

Gerber et al., 6 em 1988, descreveram a transferência do TLD para o tubérculo maior através de dupla via (superolateral e posterior), para o tratamento das LIMRs em que mesmo após a mobilização e liberação do tendão, não era possível o reparo direto no local de sua inserção. 1 Alguns autores obtiveram bons resultados no tratamento da dor e da disfunção utilizando essa técnica; 7 12 no entanto, foram encontrados 36% de falha nos casos operados utilizando a técnica de Gerber. 6 13 Há evidências de que a maioria desses insucessos ocorreram por dois motivos: deiscência da reinserção da transferência 13 e deiscência da origem do músculo deltoide devido à tensão da transferência e da espessura do TLD. 16 Miyazaki et al., 11 em 2019, descreveram a técnica da transferência do TLD com alongamento utilizando enxerto homólogo para o tratamento das LIMRs através de modificações à técnica original de Gerber et al., 6 na tentativa de evitar essas duas complicações simultaneamente. Propuseram as seguintes modificações: o TLD é alongado e reforçado com um enxerto tendíneo homólogo proveniente de banco de tecidos, sendo realizada a transferência por uma única via deltopeitoral. 11 Quando comparadas as duas técnicas, Checchia et al. 17 evidenciaram, a médio prazo, que os resultados funcionais da técnica modificada por Miyazaki et al. 18 foram superiores aos da técnica original de Gerber et al. 6

No entanto, há dificuldades na obtenção dos enxertos homólogos devido aos seus custos e escassez de doadores. Imai et al. 19 utilizaram enxerto autólogo de TFL no tratamento de 39 pacientes com LIMRs associadas à pseudoparalisia, demonstrando que, o uso deste tipo de enxerto seria uma boa opção. Pochini et al. 20 utilizaram também enxerto autólogo de TFL retirada por técnica minimamente invasiva como reforço para reconstrução de lesões do peitoral maior, demonstrando a possibilidade desta opção nas reconstruções tendíneas.

O objetivo primário deste trabalho foi avaliar os resultados funcionais dos pacientes submetidos à transferência do TLD alongado e reforçado com um enxerto autólogo de TFL para tratamento das LPSIMRs. O objetivo secundário foi avaliar as possíveis complicações da retirada do enxerto da coxa.

## Métodos

Este é um estudo retrospectivo em que foram avaliados os dados clínicos e epidemiológicos, nos períodos pré e pós-operatório, de 21 pacientes com diagnóstico de LIMRs, submetidos à transferência do TLD, alongado com enxerto tendíneo autólogo de TFL, retirado da coxa contralateral à lesão do ombro, para tratamento de LPSIMRs, durante o período de novembro de 2020 a julho de 2023. Como dois pacientes se negaram a participar do estudo e dois outros não completaram o período mínimo de reabilitação após o procedimento cirúrgico, ao final, restaram 17 pacientes. As lesões foram diagnosticadas por meio do exame clínico e da ressonância magnética (RM) e confirmadas no período intraoperatório.

Os critérios de inclusão foram pacientes acima de 18 anos de idade com LIMRs sendo submetidos à técnica descrita, que aderiram ao protocolo de reabilitação, com seguimento mínimo de 1 ano. Os critérios de exclusão foram: não consentimento à participação do estudo e não adesão ao protocolo de reabilitação.

Dos 17 pacientes operados, 8 (47%) eram do sexo masculino e 9 (53%) do sexo feminino, com média de idade no momento da cirurgia de 62,6 (54–73) anos, e 13 (76,5%) cirurgias ocorreram nos ombros dominantes. A etiologia foi traumática em 12 (70,6%) pacientes ( Tabela 1 ).

**Tabela 1:** Dados demográficos e pré-operatórios

Número do caso	Sexo	Idade (anos)	Dominância	Traumático	Sintomas (em meses)	Supra	Infra	Classificação de Hamada et al. 21	Acompanhamento (em meses)
1	F	64	+		24	4	2	3	45
2	F	67	+		120	3	3	2	33
3	F	57	+	+	4	3	3	2	28
4	M	66		+	20	4	3	1	26
5	M	62	+	+	5	2	3	1	25
6	M	70	+	+	12	2	2	2	25
7	F	55	+	+	7	4	2	3	23
8	F	73	+	+	72	3	3	1	19
9	M	54			72	4	4	4A	17
10	F	54		+	10	4	4	1	17
11	M	57	+	+	20	4	4	3	16
12	M	61		+	2	4	2	3	16
13	M	65	+	+	6	3	1	1	15
14	F	58	+	+	120	4	2	3	15
15	F	70	+		24	4	4	4A	15
16	M	65	+		12	3	4	1	13
17	F	66	+	+	4	3	2	2	12
Média		62,6			31,4				21,2

**Abreviaturas** : F, feminino; Infra, músculo infraespinal; M, masculino; Supra, músculo supraespinal.

O período médio entre o aparecimento dos sintomas e da cirurgia foi de 31,4 (2–120) meses, com um período médio de acompanhamento pós-operatório de 21,2 (12–45) meses ( Tabela 1 ).

Todos os pacientes foram submetidos a radiografias do ombro acometido, em que foram avaliados sinais de artropatia do manguito rotador classificados por Hamada et al. 21 Foram ainda submetidos ao exame de RM, no qual, além da lesão, foi avaliada a infiltração gordurosa dos músculos supra e infraespinal, que foi classificada de acordo com o método descrito por Goutallier e modificado por Fuchs et al. 22 ( Tabela 1 )

Seis pacientes foram classificados como grau 1, 4 como grau 2, 5 como grau 3 e 2 como grau 4A, de acordo com a classificação de Hamada et al. 21 ( Tabela 1 ).

Segundo a classificação de Goutallier modificada por Fuchs et al., 22 2 casos eram grau II, 6 grau III e 9 grau IV do músculo supraespinal e 1 caso grau I, 6 casos grau II, 5 grau III e 5 grau IV do músculo infraespinal ( Tabela 1 ).

**Fig. 1:**
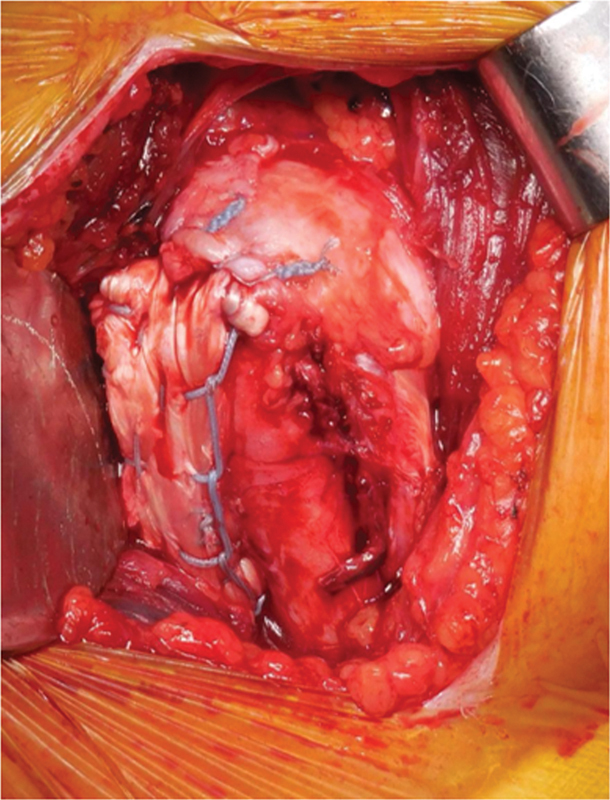


A técnica utilizada para transferência do TLD com enxerto e o protocolo de reabilitação pós-operatória foram descritos por Checchia et al., 13 com a diferença de ter sido utilizado enxerto autólogo de TFL para sua fixação na região póstero-superior do tubérculo maior ( Fig. 1 ).

**Fig. 2:**
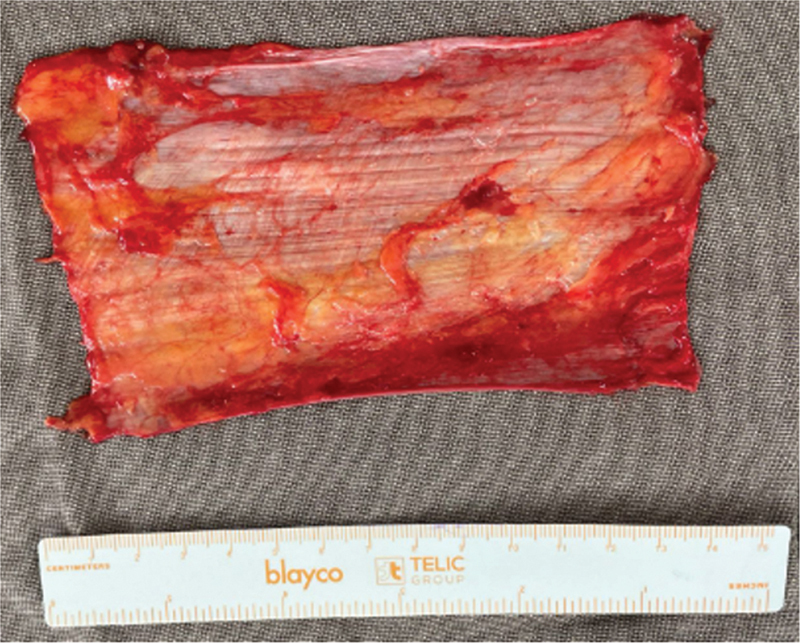


**Fig. 3:**
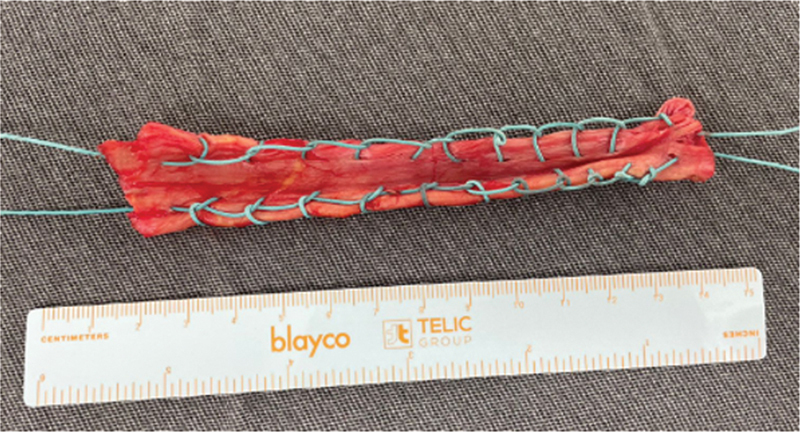


**Fig. 4:**
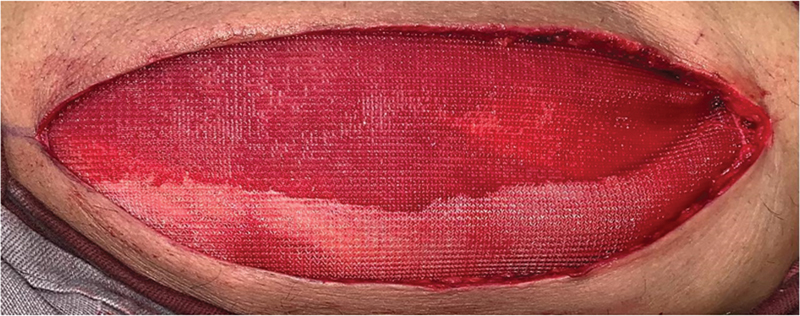


O enxerto autólogo de TFL foi retirado da face anterolateral da coxa contralateral à lesão, através de uma via com aproximadamente 15 cm de extensão, sendo retirado um enxerto com dimensões de 15 cm de comprimento por 5 cm de largura ( Fig. 2 ), sendo preparado dobrando o enxerto ao meio e reforçado com fios de poliéster número 2 ( Fig. 3 ). Após a retirada, foi utilizada uma tela cirúrgica de polipropileno para o fechamento da ferida ( Fig. 4 ).

A área doadora do enxerto foi avaliada analisando se houve alteração na força de abdução do quadril, quando comparado ao contralateral, além de possíveis complicações como infecções, dor local, hérnias incisionais e contratura em adução, através do teste de Ober. A força de abdução do quadril foi aferida nos dois membros utilizando um dinamômetro devidamente calibrado. As forças foram comparadas avaliando se houve ou não perda de força do membro da coxa doadora do enxerto.

As amplitudes de movimento (ADM) ativa nos períodos pré e pós-operatório foram registradas de acordo com o método proposto pela *American Academy of Orthopaedic Surgeons* . 23 Os resultados funcionais foram avaliados de acordo com o sistema de pontuação da University of California, Los Angeles (UCLA) idealizado por Ellman et al. 24 Foram comparados estatisticamente as ADMs e os resultados funcionais dos períodos pré com os resultados obtidos no pós-operatório.

As variáveis contínuas do mesmo grupo foram comparadas por meio de testes t pareados. As variáveis contínuas de grupos diferentes foram comparadas por meio de testes t não pareados. As correlações entre as variáveis contínuas foram determinadas pelo cálculo do coeficiente de Pearson. Os valores de *p* ≤ 0,05 foram considerados significativos.

O trabalho foi submetido ao Comitê de Ética em Pesquisa e aprovado conforme o CAAE: 79565024.6.0000.5479.

## Resultados

A elevação ativa média foi de 96,5° no período pré-operatório e 133° no pós-operatório ( *p* < 0,05). A rotação lateral (RL) ativa média foi de 41,2° no período pré-operatório para 52,8° no pós-operatório ( *p* < 0,05). A rotação medial (RM) ativa média foi em L2 no período pré-operatório e em T9 no pós-operatório ( *p* < 0,05) ( Tabela 2 ).

**Tabela 2:** Avaliação da amplitude de movimento e dos resultados funcionais nos períodos pré e pós-operatórios

Número do caso	ADM						UCLA	
	ELEV PRÉ (°)	ELEV PÓS (°)	RL PRÉ (°)	RL PÓS (°)	RM PRÉ	RM PÓS	TOTAL PRÉ	TOTAL PÓS
1	60	160	40	60	L3	T12	8	28
2	140	100	70	60	L2	T7	13	24
3	80	160	45	60	T7	T6	7	33
4	60	100	10	40	L4	T12	15	23
5	80	160	60	60	L2	T8	10	33
6	60	100	60	60	Glúteo	L5	27	14
7	80	100	60	90	L5	T7	6	30
8	50	100	50	60	L4	T5	13	33
9	150	160	45	45	T7	T12	12	31
10	90	100	30	30	L4	T7	5	21
11	90	160	80	90	Glúteo	T5	11	33
12	80	160	10	30	T7	T5	15	28
13	140	160	30	80	L4	T7	13	33
14	100	120	20	45	L5	L2	8	19
15	140	160	30	30	T8	T8	7	32
16	150	160	30	45	T7	T5	13	31
17	90	100	30	30	Glúteo	Glúteo	8	28
Média	96,47	132,94	41,17	52,82	L2	T9	11,23	27,88
	*p* < 0,05		*p* < 0,05		*p* < 0,05		*p* < 0,05	

**Abreviaturas** : ADM, amplitude de movimento; ELEV, elevação; PÓS, pós-operatório; PRÉ, pré-operatório; RL, rotação lateral; RM, rotação medial.

A pontuação no período pré-operatório média foi de 11,2 (7–27) pontos, enquanto a pontuação média pós-operatória foi de 27,9 (14–33) pontos ( *p* < 0,05) ( Tabela 2 ). De acordo com os resultados da pontuação UCLA no período pós-operatório, 12 pacientes obtiveram resultados satisfatórios (≥ 28 pontos) e 5, resultados insatisfatórios (≤ 27 pontos). Observamos que três pacientes mantiveram a queixa de dor após o procedimento. Em um dos casos operados (caso número 6), houve piora da pontuação UCLA; porém, foi um caso de rerrotura do manguito rotador.

Em relação à área doadora, ao analisarmos a força média de abdução (FMA) do quadril, observamos que 9 pacientes (53%) apresentaram uma FMA mais baixa do local de onde foi retirado o enxerto quando comparado à FMA contralateral, 6 pacientes (35,3%) apresentaram FMA mais alta no lado do enxerto e 2 pacientes (11,76%) apresentaram FMA igual nos dois membros. No entanto, na diferença das médias de abdução dos membros dos quais o enxerto foi retirado e do membro contralateral não houve diferença estatística ( *p* = 0,578).

Em relação ao local da retirada do enxerto de TFL: nenhum paciente apresentou sinais de infecção, dois pacientes queixaram-se de dor junto à ferida operatória e um paciente apresentou uma hérnia incisional. Avaliamos também a contratura em adução do membro de onde foi retirado o enxerto através do teste de Ober, o qual nenhum paciente apresentou resultado positivo para o teste.

## Discussão

Os pacientes deste trabalho apresentaram resultados clínicos satisfatórios em 70,6% dos casos, demonstrando ser a técnica aqui descrita uma alternativa à artroplastia reversa em casos de LIMRs. Resultados semelhantes foram obtidos por Miyazaki et al. 18 e por Gerber et al., 1 que obtiveram 71% e 62% de resultados satisfatórios em seus estudos, respectivamente.

A melhora da ADM entre os períodos pré e pós-operatório foi estatisticamente significante, com ganho de 36,5° de elevação ( *p* < 0,05), 12,7° de RL ( *p* < 0,05) e de 5 níveis vertebrais de RM ( *p* < 0,05) ( Tabela 2 ). Estes resultados foram semelhantes aos observados por outros autores como Boileau et al., 9 que utilizaram a transferência do TLD para o tratamento de LIMRs em 15 pacientes, e obtiveram um ganho de 34,7° de elevação após o procedimento. Quando analisamos os resultados funcionais dos pacientes submetidos à transferência do TLD com uso de enxerto homólogo da série de Miyazaki et al., 18 observamos que seus pacientes apresentaram melhora da ADM e da pontuação UCLA, assim como foi observado nos pacientes deste estudo.

Aoki et al. 7 realizaram 12 transferências do TLD com seguimento médio de 35,6 meses, e obtiveram um ganho de 36° de elevação após o procedimento, e a avaliação funcional foi realizada através da pontuação UCLA, na qual obtiveram 75% de resultados satisfatórios com pontuação média de 28 pontos. Os mesmos resultados não foram reproduzidos por Miniaci et al., 8 que, em 17 pacientes submetidos à transferência do TLD com seguimento médio pós-operatório de 51 meses, observaram uma discreta melhora da pontuação UCLA, de 6,8 no período pré-operatório para 16,4 pontos no pós-operatório. Bouchard et al., 15 em sua revisão sistemática, avaliaram 980 pacientes submetidos a transferências tendíneas com média de idade de 58,9 anos com seguimento médio de 44,7 meses, sendo mais comum a utilização do TLD para o tratamento das LIMRs, e observaram que houve aumento de 18,7 pontos na pontuação UCLA após o procedimento.

O uso de aloenxerto dérmico acelular foi utilizado por diversos autores no reparo de LIMRs. 25 26 Gupta et al. 25 utilizaram o aloenxerto no preenchimento da falha entre o manguito rotador e seu *footprint* em 24 pacientes, com média de idade de 63 anos, com melhora da flexão e RL, além de não serem observadas complicações relacionadas ao enxerto. Skedros et al. 26 descreveram um caso clínico de transferência do LD alongado com aloenxerto dérmico acelular obtendo resultados satisfatórios de flexão ativa de 180° e RL de 60°.

Assim como nós, outros autores também utilizaram enxertos autólogos de TFL 19 27 e de banda de trato iliotibial (BTIT) 28 nos reparos das LIMRs. Mori et al. 27 compararam o uso de enxerto autólogo de TFL por via artroscópica versus reparo parcial artroscópico em 48 pacientes com LIMRs, sendo 24 pacientes de cada um desses grupos. Com seguimento médio de 35 meses, observaram que o grupo que utilizou o enxerto apresentou melhores resultados funcionais e menor taxa de rerrotura (8,3%) quando comparado ao grupo que foi submetido ao reparo artroscópico parcial, cuja taxa de rerrotura foi de 41,7%. 27 Mihara et al. 28 observaram que o uso da BTIT com plug ósseo no reparo das LIMRs, com seguimento de 2 anos, não levou a nenhum caso de rerrotura do enxerto. Imai et al. 19 utilizaram enxerto autólogo de TFL em 39 pacientes com LIMRs associadas a pseudoparalisia, com idade inferior a 60 anos, obtendo melhora da elevação de 57° para 131° e RL de 17° para 32°, sendo uma opção viável para pacientes jovens com pseudoparalisia.

Lewington et al., 29 em sua revisão sistemática, compararam 15 estudos que utilizaram diferentes tipos de enxertos nos reparos das LIMR e observaram que podem apresentar resultados funcionais equivalentes; porém, o uso do enxerto autólogo estaria associado à maiores riscos de morbidade e ao aumento do tempo cirúrgico. No nosso estudo, observamos que a retirada do enxerto de TFL não trouxe complicações relevantes para a área doadora e não houve aumento do tempo cirúrgico, uma vez que, a retirada do enxerto era realizada no mesmo momento em que foi realizado a abordagem do ombro acometido, pois disponibilizamos de duas equipes para atuar simultaneamente no procedimento cirúrgico. Observamos também em nosso estudo que quando comparada a força do membro inferior da área doadora, não houve perda de força do quadril em relação ao membro inferior contralateral. O uso de enxerto tendíneo homólogo, pode representar desvantagem a longo prazo, uma vez que há preocupação com sua sobrevida. Há evidências de que o uso de enxertos homólogos apresentou taxas de falhas superiores às dos enxertos autólogos. 30 Getgood et al. 30 compararam o uso dos enxertos autólogo e homólogo na reconstrução do ligamento cruzado anterior, e observaram que a taxa de rerrotura é três vezes superior nos pacientes tratados com enxerto homólogo.

Outro fator limitante em relação ao uso dos enxertos homólogos é a necessidade de cadastramento do médico cirurgião e do serviço hospitalar junto ao Ministério da Saúde para que o enxerto possa ser fornecido e utilizado. Após o cadastramento, deve-se checar a disponibilidade do enxerto solicitado sendo baixo a disponibilidade devido à escassez de doadores aos bancos de tecidos. Devido à baixa disponibilidade, necessidade de cadastramento e logística para a obtenção desses enxertos, o uso do enxerto autólogo torna-se uma opção viável e com baixos índices de complicações.

Este estudo apresenta algumas limitações, tais como a não-avaliação da força dos períodos pré e pós-operatórios, pois os pacientes foram avaliados apenas pela pontuação UCLA, assim como o curto período de seguimento pós-operatório, sendo que, na literatura, os estudos apresentaram um seguimento mais longo, com mínimo de 24 meses.

## Conclusão

Neste estudo, observamos que após um período mínimo de 12 meses, a técnica realizada no tratamento de lesões posterossuperiores irreparáveis do manguito rotador foi de 70,6% de resultados satisfatórios de acordo com a pontuação UCLA e melhora da elevação e rotações lateral e medial. O uso do enxerto autólogo é uma opção viável com índices de complicações de 17,6% da amostra, tanto no ombro quanto no local de onde foi retirado o enxerto.
